# Impact of the Mode of Extraction on the Lipidomic Profile of Oils Obtained from Selected Amazonian Fruits

**DOI:** 10.3390/biom9080329

**Published:** 2019-08-01

**Authors:** Juliana Erika Cristina Cardona Jaramillo, Marcela Piedad Carrillo Bautista, Oscar Alberto Alvarez Solano, Luke E. K. Achenie, Andrés Fernando González Barrios

**Affiliations:** 1 Grupo de Diseño de Productos y Procesos (GDPP), Department of Chemical Engineering, Universidad de los Andes. Carrera 1E No. 19 A 40 Edificio Mario Laserna, 19A-40 Bogotá, Colombia; 2 Instituto Amazónico de Investigaciones Científicas Sinchi. Calle 20 # 5-44 Bogotá, Colombia; 3 Multiscale and Multiphysics Modeling Lab, Department of Chemical Engineering, Virginia Tech (Virginia Polytechnic Institute and State University), 298 Goodwin Hall, Blacksburg, VA 24060, USA

**Keywords:** oil extraction, lipidomic profile, Amazonian palms.

## Abstract

Oils and fats are important raw materials in food products, animal feed, cosmetics, and pharmaceuticals among others. The market today is dominated by oils derive, d from African palm, soybean, oilseed and animal fats. Colombia’s Amazon region has endemic palms such as *Euterpe precatoria* (açai)*, Oenocarpus bataua* (patawa)*,* and *Mauritia flexuosa* (buriti) which grow in abundance and produce a large amount of ethereal extract. However, as these oils have never been used for any economic purpose, little is known about their chemical composition or their potential as natural ingredients for the cosmetics or food industries. In order to fill this gap, we decided to characterize the lipids present in the fruits of these palms. We began by extracting the oils using mechanical and solvent-based approaches. The oils were evaluated by quantifying the quality indices and their lipidomic profiles. The main components of these profiles were triglycerides, followed by diglycerides, fatty acids, acylcarnitine, ceramides, ergosterol, lysophosphatidylcholine, phosphatidyl ethanolamine, and sphingolipids. The results suggest that solvent extraction helped increase the diglyceride concentration in the three analyzed fruits. Unsaturated lipids were predominant in all three fruits and triolein was the most abundant compound. Characterization of the oils provides important insights into the way they might behave as potential ingredients of a range of products. The sustainable use of these oils may have considerable economic potential.

## 1. Introduction

There is a growing tendency to include natural, often new, ingredients in mass consumer products. This is particularly true in the cosmetics and food industries, where these ingredients are used to continually improve and enhance their products in order to compete in a highly competitive market [1]. However, the inclusion of these natural ingredients in product formulations is far from easy because they are complex mixtures and more research is required for a full understanding of their compositions [2,3,4]. This could enable substantial improvements in the design of successful products containing these ingredients [2].

There are around 2600 species of palms in the world as a whole, while in South America there are 50 genres and approximately 476 species, of which 85% are endemic to the subcontinent [5,6]. Palms play a dominant role in tropical American forests in comparison to other botanical families [7,8]. This large number of species combined with the ethnic and cultural wealth of the area has given rise to an extensive range of uses of palm products and associated management practices [5]. The most important products derived from South American palms are edible fruits, palm hearts, oils, as well as stems and leaves for construction purposes [5]. Conservation of the forests is currently being defended through the enforcement of sustainable harvesting practices [5,9].

Just like the African palm fruit, some fruits of palm species endemic to the tropical forest of the Amazon could be potential sources of oil [10]. The Instituto Amazónico de Investigaciones Científicas Sinchi in Colombia has identified the following palm species as promising: *Euterpe precatoria* (one of the two commercial species of açai together with, although less studied than, *Euterpe oleraceae* [11])*, Mauritia flexuosa* (buriti)*,* and *Oenocarpus bataua* (patawa) [12]. All of these palms grow abundantly in certain areas of Amazonia and are considered a possible source of oil [10,13]. In fact, the extracts of some of those fruits are already being used in some cosmetics and food products [14,15], and their extracts have been widely studied due to their antioxidant capacity [15,16,17,18,19,20,21,22,23,24,25,26,27]. However, before seriously considering the potential of these oils as lipid sources in a wider range of cosmetics, toiletries, or food products, it is necessary to characterize their lipid composition as accurately as possible and assess how this composition could be affected by the oil extraction procedure.

The extraction of oils from plants at a commercial scale is normally based on mechanical procedures (for example pressing [28]), which are generally less efficient than those using non-polar solvents such as petroleum benzine, hexane, or pentane [28,29]. There is a global tendency to switch to environmentally sustainable techniques [30] such as cold pressing [31,32], green solvents [33,34], enzymes [35], ultrasound [34], supercritical fluids [20,28,36,37], and microwaving [31,38]. All of these extraction techniques have advantages and disadvantages in terms of efficiency, effectiveness, and sustainability, with the result that a lot of research in this field has focused on comparing these different techniques, looking above at all at their respective yields [39,40,41]. However little research has been done on how the extraction technique can affect the composition or the physicochemical characteristics of the extracted oil [31,36]. These oils are normally characterized by assessing certain parameters, such as the oil quality indices and the fatty acid methyl esters (FAMEs) profile [33,35]. However, methods like these involve the derivatization of the sample, making it impossible to clarify whether the extraction method affects the composition of the main components of the oil: triglycerides (TAGs), diglycerides (DAGs), and free fatty acids (FAs). State-of-the-art chromatographic and spectrometric techniques can perhaps offer better ways of identifying the composition of these natural ingredients [42,43].

The main goal of this paper is to discuss the scientific aspects of the comparative analysis of the characteristics and composition of the oils (in terms of TAGs, DAGs, and FAs) extracted from açai, buriti, and patawa fruits using either a screw press or a non-polar solvent. Our results will help pave the way to understanding the effect of the extraction strategy on the behavior of these oils during the product formulation process. This characterization could therefore become the basis for the rational design of bioproducts containing these ingredients.

## 2. Materials and Methods

### 2.1. Plant Material Collection and Suitability

Fruits samples of *E. precatoria* Mart (açai), *M. flexuosa* (buriti), and *O. bataua* (patawa) were picked by staff from the “El Trueno” experimental station (Instituto Amazónico de Investigaciones Científicas Sinchi), which is located between San José del Guaviare and El Retorno (N 2°24′, W 72°43′). The fruits were picked when ripe in two different harvesting periods (one per year, in April 2017 and 2018 for açai, and October 2017 and 2018 for buriti and patawa). They were then washed and disinfected, after which the pulp was removed using a vertical stainless-steel machine (Metvisa) and dried in a convection oven at 40 °C for 24 h. The dried material was then powdered in a knife mill (Fritsch) to an average particle size of 1 mm. Finally, the powder was stored in a cool, dry place until the oil was extracted. 

### 2.2. Extraction of Oil from Selected Palms Species

Mechanical extraction of 500 g of dry material was performed using an expeller press at laboratory scale with a rotational speed of 60 rpm, a temperature of 25–30 °C, and a diameter of 8 mm at the extruded exit. The second extraction method involved the exhaustive extraction of oil from the material at room temperature using petroleum benzine with a 40–60 °C boiling range as a solvent (500 g of plant material/500 mL of petroleum benzine). The solvent was recovered using a rotary evaporator (Heidolph) at 40 °C. All the oils were then centrifuged at 4500 rpm (25 °C/30 min, Thermo Fisher Scientific, Waltham, MA, USA), pulling apart all suspended particulate matter. Samples were extracted in triplicate.

### 2.3. Physicochemical Characterization

Oil quality indices were determined according to the official methods of the American Oil Chemists’ Society (AOCS), calculating saponification value by AOCS Cd 3-25, iodine value by AOCS Cd 1.25, and acid value by AOCS Cd 3d-63 [44]. Density was measured by a gravimetric method.

The fatty acid methyl esters (FAMEs) were analyzed by preparing samples according to ISO–5509. An amount of 0.5 g of each oil was saponified with 6 mL of a 0.5 M methanolic solution of NaOH with reflux at 90 °C until the oil droplets disappeared. Then, 7 mL of a methanolic solution of 14% BF_3_ was added and the mixture was boiled for 3 minutes. After that, FAMEs were extracted from the reaction mixture with isooctane. The phases were separated by adding 20 mL of a saturated solution of NaCl. The hydrophobic phase was then separated and excess water was removed by the addition of 0.2 g of anhydrous Na_2_SO_4_. The sample obtained was analyzed by gas chromatography with a flame ionization detector (GC-FID); separation was achieved using an RTX-Wax column (30m × 0.25 mm 0.5 um) of RESTEK^®^ (Belfort, PA, USA). Helium was used as the carrier gas at 40 cm^3^ min^−1^. The injector temperature was 200 °C and the detector temperature was 240 °C. The analysis was carried out, first in isothermal mode at 50 °C (5 min), then in program temperature mode from 50 °C to 240 °C at 5 °C min^−1^ and finally, in isothermal mode at 240 °C (15 min). FAMEs were identified by comparing their retention times with those identified with a FAMEs Supelco standard. Results were reported as relative concentrations [25].

The lipidomic profile of each oil was obtained by ultra-high-performance liquid chromatography (UHPLC) at the NIH West Coast Metabolomics Center. Each oil was diluted properly with CHCl_3_. The separation was achieved with a Waters Acquity UHPLC CHS C18 (100 mm × 2.1mm × 1.7 µm), and lipids were detected with an Agilent 6530 QTOF mass spectrometer with resolution R = 10,000 for positively charged lipids, and an Agilent 6550 QTOF mass spectrometer with resolution R = 20,000 for negatively charged lipids. The system was heated to 65 °C. The flow rate was set to 0.6 mL/min and the injection volume was 3 µL. The mobile phase A was a 60:40 mixture of acetonitrile and water with 10 mM of ammonium formate and 0.1% of formic acid. The mobile phase B was a 90:10 mixture of isopropyl alcohol and acetonitrile with 10 mM ammonium formate and 0.1% of formic acid. The elution gradient was 0 min 15%(B), 0–2 min 30% (B), 2–2.5 min 48% (B), 2.5–11 min 82% (B), 11–11.5 min 99% (B), 11.5–12 min 99% (B), 12–12.1 min 15% (B), 12.1–15 min 15% (B). Raw data was processed qualitatively using Agilent’s MassHunter software (CA, USA). Peak alignment was performed using Mass Profiler Professional. MS/MS information and the Lipid Blast library were used to identify the lipid compounds. Finally, the heights of the peaks were quantified using Mass Hunter Quant [45]. Based on the fact that the main functional group is common for all analyzed lipids (fatty acid chain), the peak intensities and areas obtained in each chromatographic analysis were assumed to be proportional to the lipid concentration. For this reason, results were reported as the ion peak area or as relative concentration. The latter was calculated as the relation between the area of each identified peak and the sum of areas of all identified peaks for each chromatogram [46].

### 2.4. Experimental Design and Data Analysis

A 3 × 2 factorial experimental design (three palm species and two extraction methods) was performed to test the null hypothesis that there were no differences in the composition of the oils when extracted by solvent or by screw-pressing, or between species. Statistical analysis of the data was performed in SPSS (IBM Corp. Released 2015. IBM SPSS Statistics for Windows, Version 23.0. Armonk, IBM Corp., Armonk NY, USA). A Tukey´s test was used to find significant differences between treatments at the 95% confidence level. A Shapiro–Wilk test was used to verify the normality of the data.

## 3. Results and Discussion 

Table 1 shows the extraction yield of oil from the three analyzed species. In general, patawa had the highest oil content and solvent extraction seems to be the most efficient process. If we assume for comparison purposes that 100% of the oil was recovered by the solvent extraction method, the mechanical extraction process had a 45–68% efficiency range depending on the particular species. The oil content of patawa was higher than the expected 51% content reported by Montúfar et al. [25], while the oil contents of buriti and açai were lower than reported (49.1% and 28.9% respectively [16]); some of the dissimilarity in the yields may be due to variations in solvent extraction procedures [47], inherent differences between regions and seasonal variations [48].

In general (Table 2), the acidity indices of the oils extracted by screw pressing were higher than those obtained by solvent extraction. It is possible that mechanical extraction is not sufficiently selective to the polarity of the metabolites with the result that plant extracts may include other types of compound that are not considered as lipids, such as organic acids. For their part, the iodine and saponification indices showed similar values for all three samples. This could be viewed as an indicator that the content of unsaturated lipid molecules is unaffected by the extraction procedures; however, this claim can be only verified by lipidomic profiling. The iodine index was lower than reported by Aquin et al. for buriti oil (90.00 mg I_2_/100g oil), suggesting that our buriti oil samples had a lower unsaturated lipids content.

The statistical analysis showed that the FAMEs profiles did not vary significantly when using mechanical or solvent extraction for each fruit (*p* > 0.05). We found FA chains made up of 14, 16, and 18 carbon atoms, whose structure contains 0, 1, or 2 double bonds. These results are consistent with the iodine indexes obtained by experiment. Oleic acid (C18:1) and palmitic acid (C16:0) seem to be the predominant fatty acid chains in all cases. Meanwhile, buriti and açai oils have around 75% of unsaturated FAs, while patawa oil has over 83% (Table 3). If we compare these results with the FAMEs profile reported by Mba et al. for African palm [49], there are notable differences between Amazonian palm oils and African palm oil in their palmitic (C16:0) and oleic (C18:1n9c) acid content. While the C16:0 concentration of Amazonian palms oils is lower than that of African palm oil, the C18:1n9c concentration is higher in all three Amazonian palms. In general, the total saturated FA content for Amazonian palm oils does not exceed 26%, while in African palm oil it is around 52% [49], the first big difference between these oils as potential raw materials. The most abundant FA was oleic acid, with values of between 62% to 80% for Amazonian palm oils and only 40% for African palm oil [49]. The second most abundant FA was palmitic acid, with values of 12% to 25% for the Amazonian palm oils compared with 44% in the African palm oil [49]. The content of miristoleic (C14:1), stearic (C18:0), and linolenic acids (C18:2n6c) in Amazonian palm oils did not exceed 15%. The FA compositions of açai and patawa oils seem to be similar, while buriti oil has a large amount of miristoleic acid (C14:1), which only appears in very small amounts (less than 0.5%) in açai, patawa, and African palm oils. This chemical characteristic could be a differentiating factor in the behavior of buriti oil if it were used as raw material, in that as miristoleic acid is a smaller chain FA, its presence could affect the crystallization temperature, and hence the structure, stability, and texture of the oil.

**Figure 1:**
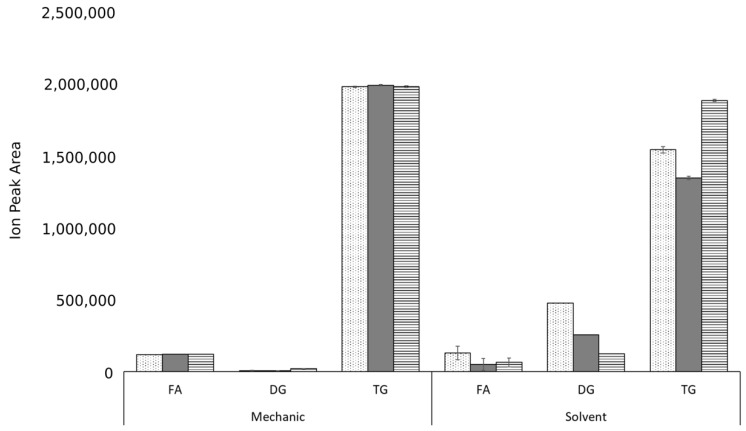
Comparison of the relative concentration of fatty acids (FA), diglycerides (DG), and triglycerides (TG) by method of extraction for açai (dotted bars), buriti (grey bars) and patawa (bars with horizontal lines) oils.

The lipidomic profile reveals that the main components are TAGs, DAGs, and FAs (Figure 1). The statistical analysis showed that the lipidomic profiles varied significantly when using mechanical or solvent extraction (*p* = 0.00). Additionally, we found there was no significant effect of interaction between the extraction method and the species analyzed, or when comparing the lipidomic profile between species (*p* < 0.05). We also identified the presence of acylcarnitine, ceramides, ergosterol, lysophosphatidyl choline, phosphatidyl ethanolamine, and sphingolipids, all of which had concentrations of less than 0.05%. The complete lipidomic profile includes approximately 458 compounds. Due to the length of the compound list, details are provided in the Supplementary Materials. These lipids are important in the formulation of toiletries and cosmetics because they are also present in the stratum corneum, the outermost layer of the epidermis, in which they act as a barrier to water loss. In aging skin, however, the production of these lipids is depleted [4]. For this reason, cosmetics and toiletries commonly include plant oils as emollients [50]. We therefore believe that açai, patawa, and buriti oils could be used as natural ingredients in products such as creams and body lotions that have great potential for moisturizing and softening skin.

As expected, the lipids with the highest concentrations in the oils were TAGs, regardless of the extraction technique (Figure 1). This is not surprising because triacylglycerols have been widely reported as the main components of plant oils and animal fats [51,52,53,54,55]. By contrast, the concentration of DAGs was affected by the type of extraction, with solvent-based extraction producing much higher concentrations. As a result, the relative concentration of TAGs was lower when solvents were used for extraction. The total FA concentration did not exceed 5%, which is consistent with the acidity index results. There was no evidence of detectable monoacylglycerol content, although this is to be expected given the metabolic pathway of plant lipids [56], in which phosphatidic acid, which has a palmitic acid chain at the *sn*-2 position, is the precursor for the synthesis of DAGs. These DAGs then help form TAGs through the diacylglycerol acyltransferase action [56].

**Figure 2:**
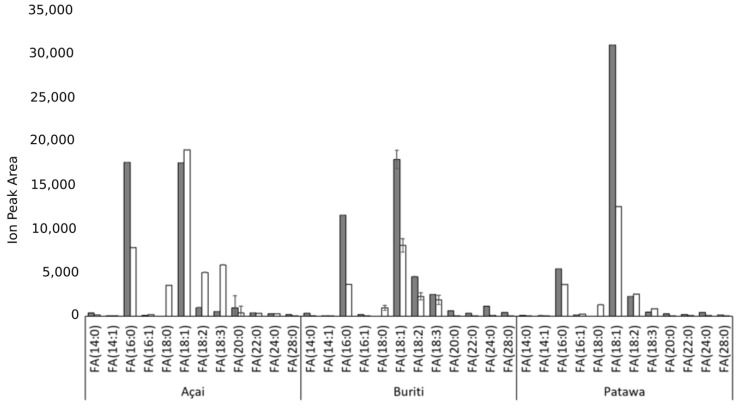
Comparison of fatty acid (FA) content according to extraction method (mechanical extraction—grey bars; solven—white bars) for açai, buriti, and patawa oils.

As regards FAs, as shown in Figure 2, mechanical extraction leads to higher FA content and, as expected, palmitic and oleic acids are the most abundant in all cases. This confirms previous reports that 16:0-CoA and 18:1-CoA are the most abundant products of plastid FA synthesis in most angiosperm species [56]. Polyunsaturated FAs were also found, albeit in lower concentrations. We noted that mechanically extracted buriti oil has FAs with longer chains. Previous reports found lignoceric acid (C24:0) to be a minor component of buriti oil [23], although, to our knowledge, this is the first time that montanic acid (C28:0) has been reported for this palm species.

**Figure 3:**
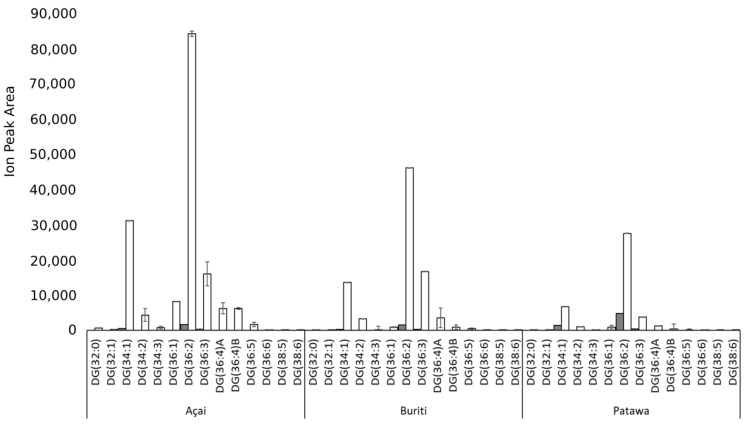
Comparison of the diglycerides (DG) content according to extraction method (mechanical extraction—grey bars; solvent—white bars) for açai, buriti, and patawa oils.

At first glance, only the solvent-extracted oils have a significant DAG content (Figure 3). The DAG content of mechanically-extracted oils was less than 1%. A higher DAG concentration in solvent-extracted oils could affect the behavior of the oil as a raw material in cosmetic and toiletry formulations as there are reports that DAGs can act as emulsifiers, which can alter the physical properties of colloid mixtures such as emulsions [57]. It is possible that the observed surface activity of DAGs makes it easier for them to be extracted by non-polar solvents than by mechanical procedures. Another possibility is that this could be a result of the chemical or enzymatic hydrolysis of TAGs, although there are few reports in the literature about this taking place in the extraction of plant oils [40].

**Figure 4:**
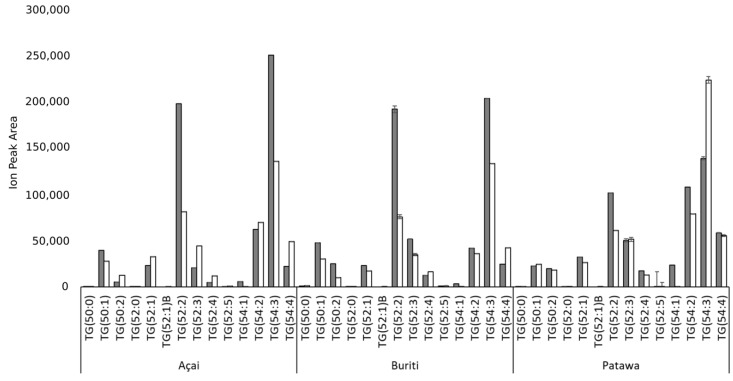
Comparison of the triglycerides (TG) content according to extraction method (mechanical extraction—grey bars; solvent—white bars) for açai, buriti, and patawa oils.

With regard to TAGs, the most abundant triacylglycerols were TG (52:2) and TG (54:3), with 52 and 54 carbon atoms, and 2 or 3 unsaturations, respectively. Both TAGs are made up of chains of palmitic or oleic acids and are consistent with the results for FAs and DAGs. Since these TAGs are the main components of the analyzed oils, we believe that these two compounds (i.e. TG (52:2) and TG (54:3)) are good models for understanding the behavior of oil as a natural ingredient in more complex systems, such as cosmetics or food products. Figure 4 shows the TAGs with a concentration of over 5%. The complete TAG profile includes 33 types of TAGs and more details can be found in the Supplementary Materials. Although buriti [21,23,58] and patawa oils [24,25] have been analyzed in previous research, this is the first time that the TAG profile for oils from these three palm species has been presented in such detail. It is also important to stress that this is the first report for oil from *E. precatoria*, one of the species of the acai plant [59].

**Figure 5:**
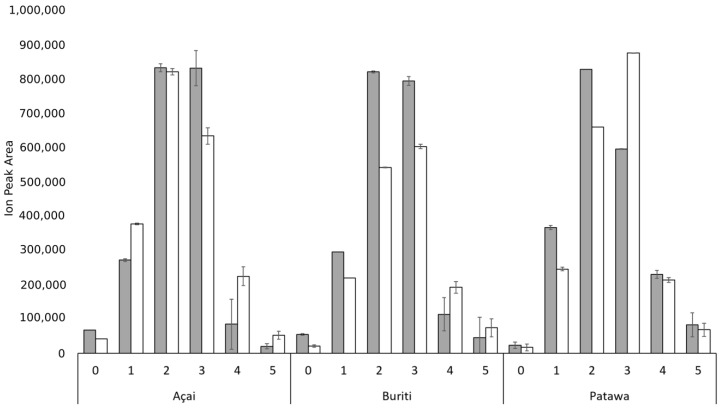
Comparison of the number of unsaturations on TAGs according to extraction method (mechanical extraction—grey bars; solvent—white bars) for açai, buriti, and patawa oils.

We noted that, while TAGs with two or three unsaturations are the most abundant in Amazonian palm oils, saturated TAGs represent less than 3% of the total weight of lipidic molecules (Figure 5). This is an important finding when it comes to understanding the behavior of Amazonian palm oil as a natural ingredient compared with that of African palm oil whose composition reveals 0, 1, or 2 unsaturation levels [49]. The concentration of TAGs with two unsaturations in Amazonian palm oils seems to be similar to that reported by Mba et al. [49] for African palm oil. However, African palm oil reached a 48% relative concentration of TAGs with only one unsaturation, while the Amazonian palm oils have up to 13% of these types of molecules. By contrast, the TAGs with three unsaturations ranged between 30% (for patawa oil) and 42% (for açai oil), but only 5% for African palm. The main difference between the Amazonian palm oils we analyzed was in the fraction of TAGs with three, four, and five unsaturations. Although açai oil had the lowest concentration of TAGs with three unsaturations, it had a larger number of TAGs with four and five unsaturations. These results appear to be consistent with the FAMEs profile described above. In this sense, Amazonian palm oils show enormous potential for use in the formulation of nutraceutical products [60]. The statistical analysis did not reveal any significant differences for the interaction between extraction methods and the specie analyzed in the TAGs profile. The samples had the same distribution in terms of the number of unsaturations on the TAGs regardless of the extraction process used. This behavior was also reported for mechanically and solvent extracted chia oils [61].

## 4. Conclusions

We performed a lipidomic profile of *E. precatoria* (açai)*, O. bataua* (patawa), and *M. flexuosa* (buriti). In general, we found that the most abundant FAs, DAGs, and TAGs are shaped by acyl chains with 16 or 18 carbons. We found significant differences between the extraction techniques, probably because the solvent extraction procedure induced a notable increase in the concentration of DAGs, leading to a significant difference in the composition of the oils extracted by solvent or by mechanical pressing. Since DAGs have surfactant activity, the oils extracted by solvent could behave in different ways from mechanically-extracted oils if they were used as natural ingredients in cosmetics, food, and toiletries formulations. We therefore believe that the extraction process is a factor worth considering before using these oils in formulations of this kind. For instance, emulsions formulated with solvent extracted oils may be more stable due to the higher DAGs content, while eco-friendly products must be formulated using mechanically extracted oils. Considering that Amazonian palms are mainly composed of unsaturated TAGs, using them as natural ingredients (regardless of the extraction procedure) could enable the production, for example, of softer cosmetic products that are more easily absorbed by the skin.

## Figures and Tables

**Table 1:** Extraction yield for açai, buriti and patawa oils obtained by mechanical and solvent extraction. (oil weight/dry material weight).

Fruit	Mechanical Extract	Solvent Extract
Açai	10%	18%
Buriti	22%	33%
Patawa	28%	62%

**Table 2:** Quality indexes for açai, buriti, and patawa oils obtained by mechanical and solvent extraction.

EXTRACTION	OIL	IODINE INDEX	SAPONIFICATION INDEX	ACIDITY INDEX %	DENSITY
MECHANICAL	Açai	68.3 ± 2.11	186.0 ± 3.1	4.83 ± 0.05	0.925 ± 0.02
	Buriti	76.4 ± 4.02	189.2 ± 0.4	6.13 ± 0.02	0.911 ± 0.04
	Patawa	76.4 ± 1.52	164.9 ± 5.1	3.92 ± 0.05	0.870 ± 0.04
SOLVENT	Açai	69.2 ± 1.89	184.0 ± 2.0	1.87 ± 0.05	0.912 ± 0.04
	Buriti	75.3 ± 2.32	187.5 ± 0.2	2.71 ± 0.02	0.910 ± 0.04
	Patawa	74.2 ± 3.40	160.7 ± 3.2	1.96 ± 0.05	0.871 ± 0.03

**Table 3:** Fatty acid methyl ester (FAME) profiles for açai, buriti and patawa oils obtained by mechanical and solvent extraction.

	Açai		Buriti		Patawa	
FAME	Mechanical	Solvent	Mechanical	Solvent	Mechanical	Solvent
C12:0	0.00%	0.80%	0.00%	0.00%	0.00%	0.16%
C14:0	0.00%	0.00%	0.00%	0.00%	0.10%	0.92%
C14:1	0.10%	1.40%	12.20%	13.61%	0.00%	0.00%
C16:0	20.50%	17.30%	25.60%	22.87%	13.00%	13.31%
C16:1	0.00%	0.00%	0.00%	0.00%	0.40%	0.00%
C18:0	4.40%	6.60%	0.00%	0.00%	3.80%	0.00%
C18:1n9c	64.70%	68.20%	62.20%	63.52%	80.00%	85.62%
C20:0	0.00%	0.00%	0.00%	0.00%	0.10%	0.00%
C18:2n6c	9.40%	4.40%	0.00%	0.00%	1.80%	0.00%
C18:3n6	0.00%	0.00%	0.00%	0.00%	0.60%	0.00%
C20:1	0.00%	0.00%	0.00%	0.00%	0.10%	0.00%
C20:2	0.30%	0.50%	0.00%	0.00%	0.00%	0.00%
C22:0	0.10%	0.10%	0.00%	0.00%	0.00%	0.00%
C20:3n6	0.00%	0.10%	0.00%	0.00%	0.00%	0.00%
SAT	25.00%	24.80%	25.60%	22.87%	17.00%	13.39%
UNS	75.00%	75.20%	74.40%	77.13%	83.00%	86.61%

## Supplementary Materials

The following are available online at https://www.mdpi.com/2218-273X/9/8/329/s1. A spreadsheet with the detailed lipidomic profile of the oils extracted from the three palm species by mechanical and solvent-based methods.
