# Supplementary material for: Impact of the Mode of Extraction on the Lipidomic Profile of Oils Obtained from Selected Amazonian Fruits
Source: Biomolecules. 2019 Aug 1;9(8):329. doi: 10.3390/biom9080329 (PMC6722717; doi:10.3390/biom9080329)
Supplement: Supplementary file 1 [file biomolecules-09-00329-s001.pdf]

	Açai		Buriti		Patawa	
Compound	Mechanical	Solvent	Mechanical	Solvent	Mechanical	Solvent
Acylcarnitine (10:0)	0.00453%	0.00000%	0.00070%	0.00000%	0.00018%	0.00000%
Acylcarnitine (10:1)	0.00000%	0.00000%	0.00000%	0.00000%	0.00000%	0.00000%
Acylcarnitine (12:0)	0.00000%	0.00010%	0.00000%	0.00005%	0.00000%	0.00002%
Acylcarnitine (12:1)	0.00000%	0.00001%	0.00000%	0.00001%	0.00000%	0.00000%
Acylcarnitine (14:1)	0.00000%	0.00000%	0.00000%	0.00000%	0.00000%	0.00000%
Acylcarnitine (14:2)	0.00000%	0.00029%	0.00000%	0.00018%	0.00000%	0.00008%
Acylcarnitine (18:1)	0.00000%	0.00005%	0.00000%	0.00006%	0.00000%	0.00001%
AcylGlcADG (50:2); AcylGlcADG (16:0-16:1-18:1);	0.00000%	0.00000%	0.00000%	0.00000%	0.00000%	0.00001%
AcylGlcADG (52:2); AcylGlcADG (16:0-18:1-18:1);	0.00000%	0.00000%	0.00000%	0.00026%	0.00000%	0.00000%
AcylGlcADG (52:3); AcylGlcADG (16:0-18:1-18:2);	0.00000%	0.00000%	0.00000%	0.00000%	0.00000%	0.00000%
AcylGlcADG (52:4); AcylGlcADG (16:1-18:1-18:2);	0.00000%	0.00000%	0.00000%	0.00000%	0.00000%	0.00000%
AcylGlcADG (54:2); AcylGlcADG (18:0-18:1-18:1);	0.00000%	0.00081%	0.00000%	0.00046%	0.00000%	0.00035%
AcylGlcADG (54:3); AcylGlcADG (18:1-18:1-18:1);	0.00000%	0.00297%	0.00000%	0.00149%	0.00000%	0.00093%
AcylGlcADG (54:4); AcylGlcADG (18:1-18:1-18:2);	0.00000%	0.00042%	0.00000%	0.00042%	0.00000%	0.00035%
AcylGlcADG (54:5); AcylGlcADG (18:1-18:2-18:2);	0.00000%	0.00000%	0.00000%	0.00000%	0.00000%	0.00000%
Ceramide (d18:1/23:0)	0.01637%	0.00000%	0.00340%	0.00000%	0.00120%	0.00000%
Ceramide (d32:1)	0.00193%	0.00000%	0.00111%	0.00000%	0.00026%	0.00000%
Ceramide (d33:1)	0.00326%	0.00000%	0.00171%	0.00000%	0.00051%	0.00000%
Ceramide (d34:0)	0.02697%	0.00160%	0.00984%	0.00004%	0.00499%	0.00001%
Ceramide (d34:1)	0.00364%	0.00032%	0.00413%	0.00042%	0.00033%	0.00008%
Ceramide (d36:1)	0.00316%	0.00004%	0.00259%	0.00015%	0.00048%	0.00004%
Ceramide (d38:1)	0.00235%	0.00009%	0.00122%	0.00011%	0.00054%	0.00008%
Ceramide (d39:1)	0.00206%	0.00000%	0.00134%	0.00000%	0.00034%	0.00000%
Ceramide (d40:0)	0.00528%	0.00000%	0.00244%	0.00000%	0.00088%	0.00000%
Ceramide (d40:1)	0.01788%	0.00008%	0.00621%	0.00009%	0.00169%	0.00008%
Ceramide (d41:1)	0.00553%	0.00005%	0.00523%	0.00008%	0.00046%	0.00007%
Ceramide (d42:1)	0.02049%	0.00007%	0.00914%	0.00016%	0.00214%	0.00012%
Ceramide (d42:2)	0.01136%	0.00000%	0.00128%	0.00000%	0.00071%	0.00000%
Ceramide (d42:2) A	0.01538%	0.00000%	0.00807%	0.00000%	0.02238%	0.00000%
Ceramide (d42:2) B	0.00280%	0.00000%	0.00119%	0.00000%	0.00148%	0.00000%
Ceramide (d43:1)	0.00369%	0.00000%	0.00235%	0.00000%	0.00040%	0.00000%
Ceramide (d44:1)	0.00399%	0.00000%	0.00216%	0.00000%	0.00046%	0.00000%
Cer-AP (t38:0); Cer-AP (t20:0/18:0);	0.00000%	0.00000%	0.00000%	0.00003%	0.00000%	0.00000%
Cer-AP (t42:0); Cer-AP (t20:0/22:0);	0.00000%	0.00012%	0.00000%	0.00098%	0.00000%	0.00001%
Cer-AP (t43:0); Cer-AP (t20:0/23:0);	0.00000%	0.00002%	0.00000%	0.00044%	0.00000%	0.00001%
Cer-AP (t44:0); Cer-AP (t20:0/24:0);	0.00000%	0.00002%	0.00000%	0.00047%	0.00000%	0.00001%
Cer-AP (t44:1); Cer-AP (t20:0/24:1);	0.00000%	0.00000%	0.00000%	0.00000%	0.00000%	0.00000%
Cer-AP (t45:0); Cer-AP (t20:0/25:0);	0.00000%	0.00000%	0.00000%	0.00000%	0.00000%	0.00001%
Cer-AP (t46:0); Cer-AP (t20:0/26:0);	0.00000%	0.00002%	0.00000%	0.00001%	0.00000%	0.00001%
Cer-AP (t46:1); Cer-AP (t20:0/26:1);	0.00000%	0.00000%	0.00000%	0.00000%	0.00000%	0.00000%
Cer-AP (t46:1); Cer-AP (t20:1/26:0);	0.00000%	0.00000%	0.00000%	0.00000%	0.00000%	0.00000%
Cer-AP (t47:0); Cer-AP (t20:0/27:0);	0.00000%	0.00000%	0.00000%	0.00000%	0.00000%	0.00000%
Cer-AP (t48:0); Cer-AP (t20:0/28:0);	0.00000%	0.00000%	0.00000%	0.00001%	0.00000%	0.00000%
Cer-AS (d36:2); Cer-AS (d18:2/18:0);	0.00000%	0.00002%	0.00000%	0.00005%	0.00000%	0.00000%
Cer-NDS (d36:0); Cer-NDS (d18:0/18:0);	0.00000%	0.00028%	0.00000%	0.00001%	0.00000%	0.00006%
Cer-NDS (d36:1); Cer-NDS (d18:0/18:1);	0.00000%	0.00185%	0.00000%	0.00000%	0.00000%	0.00001%
Cer-NDS (d36:2); Cer-NDS (d18:0/18:2);	0.00000%	0.00028%	0.00000%	0.00000%	0.00000%	0.00000%
Cer-NP (t44:0); Cer-NP (t20:0/24:0);	0.00000%	0.00001%	0.00000%	0.00000%	0.00000%	0.00001%
Cer-NP (t46:0); Cer-NP (t20:0/26:0);	0.00000%	0.00001%	0.00000%	0.00001%	0.00000%	0.00001%
Cholesteryl ester (18:1)	0.00500%	0.00000%	0.00115%	0.00000%	0.00040%	0.00000%
DG (32:0)	0.00000%	0.09504%	0.00000%	0.02351%	0.00000%	0.00643%
DG (32:1)	0.00000%	0.02567%	0.00000%	0.00870%	0.00000%	0.00759%
DG (34:1)	0.07564%	4.31188%	0.04204%	2.44140%	0.18963%	0.95679%
DG (34:2)	0.00000%	0.60107%	0.00000%	0.58088%	0.00000%	0.14666%
DG (34:3)	0.00000%	0.09750%	0.00000%	0.00869%	0.00000%	0.00839%
DG (36:1)	0.00000%	1.13213%	0.00000%	0.16045%	0.00000%	0.12410%
DG (36:2)	0.22213%	11.68015%	0.20836%	8.31026%	0.66773%	3.95312%
DG (36:3)	0.03846%	2.21568%	0.03002%	2.99761%	0.05272%	0.54974%
DG (36:4) A	0.00000%	0.86042%	0.00000%	0.62616%	0.00000%	0.17490%
DG (36:4) B	0.00000%	0.86042%	0.00000%	0.15791%	0.00000%	0.05063%
DG (36:5)	0.00000%	0.23053%	0.00000%	0.07863%	0.00000%	0.02178%

DG (36:6)	0.00000%	0.00474%	0.00000%	0.00138%	0.00000%	0.00065%
DG (38:5)	0.00000%	0.00197%	0.00000%	0.00083%	0.00000%	0.00031%
DG (38:6)	0.00000%	0.00137%	0.00000%	0.00175%	0.00000%	0.00038%
DGTS (34:1); DGTS (16:0-18:1);	0.00000%	0.02436%	0.00000%	0.04745%	0.00000%	0.03256%
DGTS (34:2); DGTS (16:1-18:1);	0.00000%	0.00451%	0.00000%	0.00727%	0.00000%	0.00608%
DGTS (34:3)	0.00000%	0.00049%	0.00000%	0.00067%	0.00000%	0.00046%
DGTS (36:3); DGTS (18:1-18:2);	0.00000%	0.00328%	0.00000%	0.00689%	0.00000%	0.00501%
Ergosterol	0.00461%	0.03268%	0.00154%	0.06323%	0.00041%	0.00796%
FA (11:0) (undecylic acid)	0.00000%	0.00006%	0.00000%	0.00006%	0.00000%	0.00004%
FA (12:0)	0.00506%	0.00000%	0.00562%	0.00000%	0.00160%	0.00000%
FA (12:0) (lauric acid)	0.00000%	0.00365%	0.00000%	0.00049%	0.00000%	0.00065%
FA (13:0) (tridecylic acid)	0.00000%	0.00010%	0.00000%	0.00007%	0.00000%	0.00005%
FA (14:0)	0.04881%	0.00000%	0.04265%	0.00000%	0.01159%	0.00000%
FA (14:0) (myristic acid)	0.00000%	0.01939%	0.00000%	0.00022%	0.00000%	0.00392%
FA (14:1)	0.00126%	0.00000%	0.00171%	0.00000%	0.00080%	0.00000%
FA (14:1) (physeteric acid)	0.00000%	0.00092%	0.00000%	0.00020%	0.00000%	0.00048%
FA (15:0)	0.01319%	0.00000%	0.01389%	0.00000%	0.01077%	0.00000%
FA (15:0) (pentadecylic acid)	0.00000%	0.01966%	0.00000%	0.00204%	0.00000%	0.01428%
FA (15:1)	0.00185%	0.00421%	0.00213%	0.00043%	0.00053%	0.00074%
FA (16:0)	2.46543%	0.00000%	1.61622%	0.00000%	0.75778%	0.00000%
FA (16:0) (palmitic acid)	0.00000%	1.08411%	0.00000%	0.64745%	0.00000%	0.51736%
FA (16:1)	0.00892%	0.00000%	0.02413%	0.00000%	0.01963%	0.00000%
FA (16:1) (palmitoleic acid)	0.00000%	0.02415%	0.00000%	0.00616%	0.00000%	0.03012%
FA (16:2)	0.00000%	0.00072%	0.00000%	0.00006%	0.00000%	0.00029%
FA (16:3)	0.00000%	0.00046%	0.00000%	0.00000%	0.00000%	0.00002%
FA (16:4)	0.00000%	0.00000%	0.00000%	0.00000%	0.00000%	0.00000%
FA (17:0)	0.07441%	0.00000%	0.05397%	0.00000%	0.01769%	0.00000%
FA (17:0) (margaric acid)	0.00000%	0.04510%	0.00000%	0.00628%	0.00000%	0.00721%
FA (17:1)	0.00586%	0.00000%	0.00584%	0.00000%	0.00415%	0.00000%
FA (17:2)	0.00000%	0.00129%	0.00000%	0.00020%	0.00000%	0.00032%
FA (18:0) (stearic acid)	0.00000%	0.49014%	0.00000%	0.17256%	0.00000%	0.18535%
FA (18:1)	2.45429%	0.00000%	2.51024%	0.00000%	4.34793%	0.00000%
FA (18:1) (oleic acid)	0.00000%	2.62710%	0.00000%	1.45246%	0.00000%	1.79673%
FA (18:2)	0.13483%	0.00000%	0.62882%	0.00000%	0.31298%	0.00000%
FA (18:2) (linoleic acid)	0.00000%	0.68990%	0.00000%	0.40313%	0.00000%	0.36338%
FA (18:3)	0.07342%	0.00000%	0.34671%	0.00000%	0.06709%	0.00000%
FA (18:3) (linolenic acid)	0.00000%	0.80923%	0.00000%	0.33172%	0.00000%	0.12057%
FA (18:4)	0.00000%	0.00017%	0.00000%	0.00000%	0.00000%	0.00005%
FA (19:0)	0.00000%	0.00504%	0.00000%	0.00084%	0.00000%	0.00072%
FA (19:1);	0.00000%	0.00250%	0.00000%	0.00085%	0.00000%	0.00071%
FA (19:2)	0.00000%	0.00013%	0.00000%	0.00002%	0.00000%	0.00002%
FA (19:3)	0.00000%	0.00018%	0.00000%	0.00001%	0.00000%	0.00000%
FA (20:0)	0.12890%	0.00000%	0.08543%	0.00000%	0.03621%	0.00000%
FA (20:0) (arachidic acid)	0.00000%	0.04997%	0.00000%	0.00427%	0.00000%	0.00681%
FA (20:1)	0.01886%	0.00000%	0.01698%	0.00000%	0.01357%	0.00000%
FA (20:1) (eicosenoic acid)	0.00000%	0.00010%	0.00000%	0.00002%	0.00000%	0.00002%
FA (20:2)	0.00234%	0.00000%	0.00352%	0.00000%	0.00141%	0.00000%
FA (20:2) (eicosadienoic acid)	0.00000%	0.00360%	0.00000%	0.00050%	0.00000%	0.00109%
FA (20:3)	0.00604%	0.00000%	0.00539%	0.00000%	0.00088%	0.00000%
FA (20:3) (eicosatrienoic acid)	0.00000%	0.00001%	0.00000%	0.00000%	0.00000%	0.00000%
FA (20:3) (homo-gamma-linolenic acid)	0.00000%	0.00657%	0.00000%	0.00026%	0.00000%	0.00014%
FA (20:4)	0.00211%	0.00000%	0.00218%	0.00000%	0.00025%	0.00000%
FA (20:4) (arachidonic acid)	0.00000%	0.00006%	0.00000%	0.00001%	0.00000%	0.00002%
FA (20:5)	0.00223%	0.00000%	0.00107%	0.00000%	0.00025%	0.00000%
FA (20:5) (eicosapentaenoic acid)	0.00000%	0.00001%	0.00000%	0.00000%	0.00000%	0.00000%
FA (21:0)	0.00000%	0.00474%	0.00000%	0.00031%	0.00000%	0.00062%
FA (21:1) A	0.00000%	0.00008%	0.00000%	0.00003%	0.00000%	0.00005%
FA (21:1) B	0.00000%	0.00006%	0.00000%	0.00003%	0.00000%	0.00005%
FA (21:4)	0.00000%	0.00047%	0.00000%	0.00075%	0.00000%	0.00053%
FA (21:5)	0.00000%	0.00001%	0.00000%	0.00000%	0.00000%	0.00001%
FA (22:0)	0.05049%	0.00000%	0.04535%	0.00000%	0.02639%	0.00000%
FA (22:0) (behenic acid)	0.00000%	0.04291%	0.00000%	0.00449%	0.00000%	0.00932%
FA (22:1)	0.00618%	0.00000%	0.00594%	0.00000%	0.00196%	0.00000%
FA (22:1) (erucic acid)	0.00000%	0.00606%	0.00000%	0.00033%	0.00000%	0.00261%

FA (22:2)	0.00139%	0.00000%	0.00075%	0.00000%	0.00033%	0.00000%
FA (22:2) (docosadienoic acid)	0.00000%	0.00036%	0.00000%	0.00001%	0.00000%	0.00042%
FA (22:4)	0.00000%	0.00007%	0.00000%	0.00008%	0.00000%	0.00008%
FA (22:5)	0.00000%	0.00001%	0.00000%	0.00000%	0.00000%	0.00000%
FA (22:6)	0.00141%	0.00000%	0.00082%	0.00000%	0.00020%	0.00000%
FA (22:6) (docosahexaenoic acid)	0.00000%	0.00000%	0.00000%	0.00000%	0.00000%	0.00000%
FA (24:0)	0.03942%	0.00000%	0.16129%	0.00000%	0.05577%	0.00000%
FA (24:0) (lignoceric acid)	0.00000%	0.03553%	0.00000%	0.01372%	0.00000%	0.01291%
FA (24:1)	0.00140%	0.00000%	0.00188%	0.00000%	0.00035%	0.00000%
FA (24:1) (nervonic acid)	0.00000%	0.00024%	0.00000%	0.00024%	0.00000%	0.00066%
FA (26:0)	0.02026%	0.00000%	0.03571%	0.00000%	0.01040%	0.00000%
FA (26:0) (cerotic acid)	0.00000%	0.00586%	0.00000%	0.00197%	0.00000%	0.00207%
FA (28:0)	0.02391%	0.00000%	0.05914%	0.00000%	0.01585%	0.00000%
FA (28:0) (montanic acid)	0.00000%	0.00601%	0.00000%	0.00259%	0.00000%	0.00174%
FAHFA (18:0); FAHFA (11:0/7:0);	0.00000%	0.00002%	0.00000%	0.00000%	0.00000%	0.00001%
FAHFA (23:1); FAHFA (18:1/5:0);	0.00000%	0.00000%	0.00000%	0.00000%	0.00000%	0.00000%
FAHFA (24:0); FAHFA (16:0/8:0);	0.00000%	0.00004%	0.00000%	0.00004%	0.00000%	0.00002%
FAHFA (24:1); FAHFA (16:1/8:0);	0.00000%	0.00001%	0.00000%	0.00000%	0.00000%	0.00001%
FAHFA (24:1); FAHFA (18:1/6:0);	0.00000%	0.00003%	0.00000%	0.00000%	0.00000%	0.00000%
FAHFA (25:0); FAHFA (16:0/9:0);	0.00000%	0.00012%	0.00000%	0.00001%	0.00000%	0.00001%
FAHFA (25:0); FAHFA (18:0/7:0);	0.00000%	0.00002%	0.00000%	0.00003%	0.00000%	0.00002%
FAHFA (25:1); FAHFA (18:1/7:0);	0.00000%	0.00002%	0.00000%	0.00000%	0.00000%	0.00000%
FAHFA (25:2); FAHFA (18:2/7:0);	0.00000%	0.00000%	0.00000%	0.00000%	0.00000%	0.00000%
FAHFA (26:0); FAHFA (10:0/16:0);	0.00000%	0.00004%	0.00000%	0.00000%	0.00000%	0.00000%
FAHFA (26:0); FAHFA (16:0/10:0);	0.00000%	0.00002%	0.00000%	0.00003%	0.00000%	0.00002%
FAHFA (26:0); FAHFA (18:0/8:0);	0.00000%	0.00004%	0.00000%	0.00003%	0.00000%	0.00002%
FAHFA (26:1); FAHFA (10:0/16:1);	0.00000%	0.00009%	0.00000%	0.00002%	0.00000%	0.00002%
FAHFA (26:1); FAHFA (18:1/8:0);	0.00000%	0.00009%	0.00000%	0.00002%	0.00000%	0.00003%
FAHFA (26:2); FAHFA (18:2/8:0);	0.00000%	0.00001%	0.00000%	0.00000%	0.00000%	0.00000%
FAHFA (27:0); FAHFA (18:0/9:0);	0.00000%	0.00004%	0.00000%	0.00002%	0.00000%	0.00001%
FAHFA (27:1); FAHFA (18:1/9:0);	0.00000%	0.00057%	0.00000%	0.00000%	0.00000%	0.00002%
FAHFA (27:2); FAHFA (18:2/9:0);	0.00000%	0.00014%	0.00000%	0.00000%	0.00000%	0.00000%
FAHFA (28:0); FAHFA (12:0/16:0);	0.00000%	0.00007%	0.00000%	0.00000%	0.00000%	0.00000%
FAHFA (28:0); FAHFA (18:0/10:0);	0.00000%	0.00001%	0.00000%	0.00000%	0.00000%	0.00000%
FAHFA (28:1); FAHFA (10:0/18:1);	0.00000%	0.00003%	0.00000%	0.00000%	0.00000%	0.00000%
FAHFA (28:1); FAHFA (12:0/16:1);	0.00000%	0.00003%	0.00000%	0.00000%	0.00000%	0.00000%
FAHFA (28:1); FAHFA (14:0/14:1);	0.00000%	0.00000%	0.00000%	0.00000%	0.00000%	0.00000%
FAHFA (28:2); FAHFA (12:0/16:2);	0.00000%	0.00008%	0.00000%	0.00029%	0.00000%	0.00019%
FAHFA (28:2); FAHFA (18:2/10:0);	0.00000%	0.00004%	0.00000%	0.00016%	0.00000%	0.00014%
FAHFA (29:1); FAHFA (18:1/11:0);	0.00000%	0.00011%	0.00000%	0.00000%	0.00000%	0.00000%
FAHFA (30:0); FAHFA (14:0/16:0);	0.00000%	0.00001%	0.00000%	0.00000%	0.00000%	0.00000%
FAHFA (30:1); FAHFA (12:0/18:1);	0.00000%	0.00000%	0.00000%	0.00000%	0.00000%	0.00000%
FAHFA (30:1); FAHFA (14:1/16:0);	0.00000%	0.00009%	0.00000%	0.00000%	0.00000%	0.00000%
FAHFA (30:1); FAHFA (16:0/14:1);	0.00000%	0.00005%	0.00000%	0.00006%	0.00000%	0.00004%
FAHFA (30:2); FAHFA (14:0/16:2);	0.00000%	0.00008%	0.00000%	0.00000%	0.00000%	0.00000%
FAHFA (30:2); FAHFA (14:1/16:1);	0.00000%	0.00014%	0.00000%	0.00000%	0.00000%	0.00000%
FAHFA (30:3); FAHFA (14:1/16:2);	0.00000%	0.00002%	0.00000%	0.00000%	0.00000%	0.00000%
FAHFA (31:0); FAHFA (15:0/16:0);	0.00000%	0.00000%	0.00000%	0.00000%	0.00000%	0.00000%
FAHFA (31:1); FAHFA (15:0/16:1);	0.00000%	0.00000%	0.00000%	0.00000%	0.00000%	0.00000%
FAHFA (32:0); FAHFA (16:0/16:0);	0.00000%	0.00004%	0.00000%	0.00000%	0.00000%	0.00001%
FAHFA (32:1); FAHFA (14:0/18:1);	0.00000%	0.00000%	0.00000%	0.00000%	0.00000%	0.00000%
FAHFA (32:1); FAHFA (16:1/16:0);	0.00000%	0.00005%	0.00000%	0.00001%	0.00000%	0.00000%
FAHFA (32:2); FAHFA (14:1/18:1);	0.00000%	0.00000%	0.00000%	0.00000%	0.00000%	0.00000%
FAHFA (32:2); FAHFA (16:0/16:2);	0.00000%	0.00000%	0.00000%	0.00000%	0.00000%	0.00000%
FAHFA (32:2); FAHFA (16:1/16:1);	0.00000%	0.00000%	0.00000%	0.00000%	0.00000%	0.00000%
FAHFA (32:2); FAHFA (18:1/14:1);	0.00000%	0.00002%	0.00000%	0.00000%	0.00000%	0.00000%
FAHFA (32:3); FAHFA (16:1/16:2);	0.00000%	0.00043%	0.00000%	0.00000%	0.00000%	0.00000%
FAHFA (32:4); FAHFA (16:3/16:1);	0.00000%	0.00007%	0.00000%	0.00001%	0.00000%	0.00001%
FAHFA (32:5); FAHFA (16:3/16:2);	0.00000%	0.00000%	0.00000%	0.00000%	0.00000%	0.00000%
FAHFA (32:5); FAHFA (16:4/16:1);	0.00000%	0.00011%	0.00000%	0.00000%	0.00000%	0.00000%
FAHFA (33:0); FAHFA (17:0/16:0);	0.00000%	0.00000%	0.00000%	0.00000%	0.00000%	0.00000%
FAHFA (33:1); FAHFA (17:1/16:0);	0.00000%	0.00001%	0.00000%	0.00000%	0.00000%	0.00000%
FAHFA (33:2); FAHFA (18:1/15:1);	0.00000%	0.00000%	0.00000%	0.00000%	0.00000%	0.00000%
FAHFA (33:3); FAHFA (17:1/16:2);	0.00000%	0.00000%	0.00000%	0.00000%	0.00000%	0.00000%

FAHFA (34:0); FAHFA (18:0/16:0);	0.00000%	0.00016%	0.00000%	0.00000%	0.00000%	0.00000%
FAHFA (34:1); FAHFA (18:1/16:0);	0.00000%	0.00010%	0.00000%	0.00001%	0.00000%	0.00002%
FAHFA (34:2); FAHFA (16:1/18:1);	0.00000%	0.00002%	0.00000%	0.00000%	0.00000%	0.00000%
FAHFA (34:2); FAHFA (18:2/16:0);	0.00000%	0.00000%	0.00000%	0.00000%	0.00000%	0.00000%
FAHFA (34:3); FAHFA (16:1/18:2);	0.00000%	0.00001%	0.00000%	0.00000%	0.00000%	0.00000%
FAHFA (34:3); FAHFA (18:1/16:2);	0.00000%	0.00021%	0.00000%	0.00005%	0.00000%	0.00000%
FAHFA (34:3); FAHFA (18:3/16:0);	0.00000%	0.00001%	0.00000%	0.00002%	0.00000%	0.00000%
FAHFA (34:4); FAHFA (18:2/16:2);	0.00000%	0.00000%	0.00000%	0.00000%	0.00000%	0.00000%
FAHFA (34:5); FAHFA (18:3/16:2);	0.00000%	0.00000%	0.00000%	0.00000%	0.00000%	0.00000%
FAHFA (35:1); FAHFA (17:0/18:1);	0.00000%	0.00000%	0.00000%	0.00000%	0.00000%	0.00000%
FAHFA (35:1); FAHFA (18:1/17:0);	0.00000%	0.00000%	0.00000%	0.00000%	0.00000%	0.00000%
FAHFA (35:2); FAHFA (17:1/18:1);	0.00000%	0.00000%	0.00000%	0.00000%	0.00000%	0.00000%
FAHFA (35:2); FAHFA (18:1/17:1);	0.00000%	0.00000%	0.00000%	0.00000%	0.00000%	0.00000%
FAHFA (35:3); FAHFA (18:2/17:1);	0.00000%	0.00000%	0.00000%	0.00000%	0.00000%	0.00000%
FAHFA (36:0); FAHFA (18:0/18:0);	0.00000%	0.00002%	0.00000%	0.00000%	0.00000%	0.00000%
FAHFA (36:1); FAHFA (18:1/18:0);	0.00000%	0.00032%	0.00000%	0.00001%	0.00000%	0.00000%
FAHFA (36:2); FAHFA (18:1/18:1);	0.00000%	0.00003%	0.00000%	0.00001%	0.00000%	0.00001%
FAHFA (36:3); FAHFA (18:1/18:2);	0.00000%	0.00042%	0.00000%	0.00007%	0.00000%	0.00014%
FAHFA (36:4); FAHFA (18:1/18:3);	0.00000%	0.00067%	0.00000%	0.00004%	0.00000%	0.00003%
FAHFA (36:4); FAHFA (18:2/18:2);	0.00000%	0.00059%	0.00000%	0.00008%	0.00000%	0.00004%
FAHFA (36:5); FAHFA (18:3/18:2);	0.00000%	0.00020%	0.00000%	0.00000%	0.00000%	0.00000%
FAHFA (36:6); FAHFA (18:3/18:3);	0.00000%	0.00023%	0.00000%	0.00001%	0.00000%	0.00000%
FAHFA (38:2); FAHFA (18:1/20:1);	0.00000%	0.00000%	0.00000%	0.00000%	0.00000%	0.00000%
FAHFA (38:4); FAHFA (18:2/20:2);	0.00000%	0.00000%	0.00000%	0.00000%	0.00000%	0.00000%
FAHFA (38:4); FAHFA (20:3/18:1);	0.00000%	0.00001%	0.00000%	0.00000%	0.00000%	0.00000%
FAHFA (38:5); FAHFA (20:4/18:1);	0.00000%	0.00000%	0.00000%	0.00000%	0.00000%	0.00000%
FAHFA (40:4); FAHFA (16:0/24:4);	0.00000%	0.00002%	0.00000%	0.00000%	0.00000%	0.00000%
FAHFA (40:7); FAHFA (22:6/18:1);	0.00000%	0.00000%	0.00000%	0.00000%	0.00000%	0.00000%
FAHFA (42:1); FAHFA (18:1/24:0);	0.00000%	0.00055%	0.00000%	0.00000%	0.00000%	0.00005%
FAHFA (42:5); FAHFA (18:1/24:4);	0.00000%	0.00000%	0.00000%	0.00000%	0.00000%	0.00000%
FAHFA (42:6); FAHFA (18:2/24:4);	0.00000%	0.00000%	0.00000%	0.00000%	0.00000%	0.00000%
FAHFA (44:1); FAHFA (26:0/18:1);	0.00000%	0.00000%	0.00000%	0.00000%	0.00000%	0.00000%
GlcADG (32:1); GlcADG (14:0-18:1);	0.00000%	0.00000%	0.00000%	0.00000%	0.00000%	0.00000%
GlcADG (34:1); GlcADG (16:0-18:1);	0.00000%	0.00103%	0.00000%	0.00230%	0.00000%	0.00039%
GlcADG (34:2); GlcADG (16:1-18:1);	0.00000%	0.00000%	0.00000%	0.00000%	0.00000%	0.00000%
GlcADG (35:1); GlcADG (17:0-18:1);	0.00000%	0.00000%	0.00000%	0.00000%	0.00000%	0.00000%
GlcADG (35:2); GlcADG (17:1-18:1);	0.00000%	0.00110%	0.00000%	0.00030%	0.00000%	0.00010%
GlcADG (36:1); GlcADG (18:0-18:1);	0.00000%	0.00000%	0.00000%	0.00000%	0.00000%	0.00000%
GlcADG (36:1); GlcADG 18:0-18:1;	0.00000%	0.00212%	0.00000%	0.00306%	0.00000%	0.00137%
GlcADG (36:2); GlcADG (18:1-18:1);	0.00000%	0.00201%	0.00000%	0.00416%	0.00000%	0.00133%
GlcADG (36:3); GlcADG (18:1-18:2);	0.00000%	0.00066%	0.00000%	0.00047%	0.00000%	0.00000%
GlcCer(d40:1)	0.00000%	0.00000%	0.00000%	0.00000%	0.00000%	0.00015%
GlcCer(d42:1)	0.00000%	0.00000%	0.00000%	0.00226%	0.00000%	0.00177%
GlcCer(d42:2)	0.00000%	0.00043%	0.00000%	0.00083%	0.00000%	0.00064%
LDGTS (15:0)	0.00000%	0.00000%	0.00000%	0.00000%	0.00000%	0.00000%
LDGTS (16:3);	0.00000%	0.00000%	0.00000%	0.00000%	0.00000%	0.00000%
LDGTS (18:1);	0.00000%	0.00000%	0.00000%	0.00000%	0.00000%	0.00000%
LPC (14:0)	0.00000%	0.00000%	0.00000%	0.00000%	0.00000%	0.00000%
LPC (16:0)	0.00907%	0.00000%	0.00234%	0.00000%	0.00069%	0.00000%
LPC (16:1)	0.00374%	0.00000%	0.00129%	0.00000%	0.00025%	0.00000%
LPC (17:1)	0.00183%	0.00000%	0.00043%	0.00000%	0.00013%	0.00000%
LPC (18:0)	0.00544%	0.00000%	0.00130%	0.00000%	0.00016%	0.00000%
LPC (18:0) A	0.00000%	0.00000%	0.00000%	0.00000%	0.00000%	0.00000%
LPC (18:0) B	0.00106%	0.00000%	0.00033%	0.00000%	0.00014%	0.00000%
LPC (18:1)	0.01155%	0.00000%	0.00349%	0.00000%	0.00084%	0.00002%
LPC (18:1);	0.00000%	0.00000%	0.00000%	0.00000%	0.00000%	0.00000%
LPC (18:2)	0.00098%	0.00000%	0.00055%	0.00000%	0.00009%	0.00000%
LPC (18:2) A	0.00329%	0.00000%	0.00092%	0.00000%	0.00039%	0.00000%
LPC (18:2) B	0.00738%	0.00000%	0.00114%	0.00000%	0.00040%	0.00000%
LPC (18:3)	0.00000%	0.00000%	0.00000%	0.00000%	0.00000%	0.00000%
LPC (20:1)	0.00000%	0.00000%	0.00000%	0.00000%	0.00000%	0.00000%
LPC (20:2)	0.00331%	0.00000%	0.00056%	0.00000%	0.00041%	0.00000%
LPC (20:3)	0.00000%	0.00000%	0.00000%	0.00000%	0.00000%	0.00000%
LPC (20:4)	0.00231%	0.00000%	0.00035%	0.00000%	0.00022%	0.00000%

LPE (16:0)	0.00000%	0.00000%	0.00000%	0.00000%	0.00000%	0.00000%
LPE (16:1);	0.00000%	0.00000%	0.00000%	0.00000%	0.00000%	0.00000%
LPE (18:0)	0.00000%	0.00000%	0.00000%	0.00000%	0.00000%	0.00000%
LPE (18:2)	0.00000%	0.00000%	0.00000%	0.00000%	0.00000%	0.00000%
LPE(18:0)	0.00000%	0.00018%	0.00000%	0.00025%	0.00000%	0.00020%
LPE(18:2)	0.00000%	0.00017%	0.00000%	0.00000%	0.00000%	0.00000%
LPI (16:0);	0.00000%	0.00000%	0.00000%	0.00000%	0.00000%	0.00000%
LPI (18:0)	0.00000%	0.00000%	0.00000%	0.00000%	0.00000%	0.00000%
LPI (18:1);	0.00000%	0.00000%	0.00000%	0.00000%	0.00000%	0.00000%
LPS (18:0);	0.00000%	0.00000%	0.00000%	0.00000%	0.00000%	0.00000%
LPS (18:1);	0.00000%	0.00000%	0.00000%	0.00000%	0.00000%	0.00000%
OxFA (18:0+1O);	0.00000%	0.00107%	0.00000%	0.00010%	0.00000%	0.00012%
OxFA (18:0+2O);	0.00000%	0.00017%	0.00000%	0.00008%	0.00000%	0.00004%
OxFA (18:1+1O);	0.00000%	0.00206%	0.00000%	0.00041%	0.00000%	0.00083%
OxFA (18:1+2O);	0.00000%	0.00013%	0.00000%	0.00003%	0.00000%	0.00005%
OxFA (18:2+1O);	0.00000%	0.00599%	0.00000%	0.00046%	0.00000%	0.00287%
OxFA (18:2+2O);	0.00000%	0.00037%	0.00000%	0.00098%	0.00000%	0.00065%
OxFA (18:3+1O);	0.00000%	0.00001%	0.00000%	0.00000%	0.00000%	0.00000%
OxFA (20:3+2O);	0.00000%	0.00003%	0.00000%	0.00001%	0.00000%	0.00002%
OxFA (20:4+1O);	0.00000%	0.00003%	0.00000%	0.00000%	0.00000%	0.00001%
OxFA (20:4+4O(1Cyc));	0.00000%	0.00033%	0.00000%	0.00000%	0.00000%	0.00000%
OxFA (22:6+1O);	0.00000%	0.00000%	0.00000%	0.00000%	0.00000%	0.00000%
PA (34:1); PA (16:0-18:1);	0.00000%	0.00000%	0.00000%	0.00000%	0.00000%	0.00000%
PA (36:2); PA (18:1-18:1);	0.00000%	0.00009%	0.00000%	0.00000%	0.00000%	0.00028%
PA (36:3); PA (18:1-18:2);	0.00000%	0.00000%	0.00000%	0.00013%	0.00000%	0.00000%
PA (36:4); PA (18:2-18:2);	0.00000%	0.00008%	0.00000%	0.00000%	0.00000%	0.00000%
PC (16:0/9:0(CHO))	0.00000%	0.00026%	0.00000%	0.00072%	0.00000%	0.00042%
PC (28:0)	0.00267%	0.00000%	0.00112%	0.00000%	0.00024%	0.00000%
PC (30:0)	0.00277%	0.00000%	0.00095%	0.00000%	0.00037%	0.00000%
PC (30:1)	0.00346%	0.00000%	0.00089%	0.00000%	0.00035%	0.00000%
PC (32:0)	0.01195%	0.00000%	0.00180%	0.00000%	0.00089%	0.00000%
PC (32:1)	0.01155%	0.00116%	0.00300%	0.00112%	0.00115%	0.00076%
PC (32:2)	0.00474%	0.00000%	0.00084%	0.00071%	0.00038%	0.00084%
PC (33:0)	0.00000%	0.00058%	0.00000%	0.00054%	0.00000%	0.00000%
PC (33:1)	0.00777%	0.00162%	0.00124%	0.00070%	0.00051%	0.00061%
PC (33:2)	0.00467%	0.00000%	0.00122%	0.00000%	0.00036%	0.00000%
PC (34:0)	0.00611%	0.00176%	0.00121%	0.00137%	0.00054%	0.00057%
PC (34:1)	0.09834%	0.00635%	0.00992%	0.01612%	0.00219%	0.00661%
PC (34:2)	0.06288%	0.00840%	0.00775%	0.02549%	0.00247%	0.01187%
PC (34:3)	0.01846%	0.00000%	0.00289%	0.00000%	0.00055%	0.00000%
PC (34:3) A	0.00000%	0.00067%	0.00000%	0.00136%	0.00000%	0.00074%
PC (34:3) B	0.00000%	0.00067%	0.00000%	0.00136%	0.00000%	0.00074%
PC (34:3) C	0.00000%	0.00048%	0.00000%	0.00116%	0.00000%	0.00044%
PC (34:4)	0.00373%	0.00000%	0.00051%	0.00000%	0.00025%	0.00000%
PC (35:1)	0.00738%	0.00085%	0.00178%	0.00835%	0.00055%	0.00075%
PC (35:2)	0.01046%	0.00000%	0.00319%	0.00000%	0.00086%	0.00000%
PC (35:2) A	0.00000%	0.00239%	0.00000%	0.00495%	0.00000%	0.00098%
PC (35:2) B	0.00000%	0.00239%	0.00000%	0.00495%	0.00000%	0.00098%
PC (35:3)	0.00294%	0.00096%	0.00119%	0.00589%	0.00037%	0.00185%
PC (35:4)	0.00000%	0.00073%	0.00000%	0.00160%	0.00000%	0.00100%
PC (36:1)	0.02338%	0.00401%	0.00851%	0.00880%	0.00130%	0.00542%
PC (36:2)	0.12809%	0.01135%	0.03891%	0.02420%	0.00516%	0.01435%
PC (36:3) A	0.04060%	0.00387%	0.01507%	0.00745%	0.00139%	0.00448%
PC (36:3) B	0.00498%	0.00186%	0.00258%	0.00686%	0.00028%	0.00335%
PC (36:4) A	0.02711%	0.00131%	0.00524%	0.00289%	0.00086%	0.00159%
PC (36:4) B	0.00000%	0.00000%	0.00000%	0.00530%	0.00000%	0.00053%
PC (36:4) C	0.00000%	0.00034%	0.00000%	0.00731%	0.00000%	0.00295%
PC (36:5) A	0.00768%	0.00000%	0.00117%	0.00000%	0.00029%	0.00000%
PC (37:2)	0.01200%	0.00172%	0.00272%	0.01311%	0.00091%	0.00178%
PC (37:3)	0.00000%	0.00252%	0.00000%	0.00578%	0.00000%	0.00031%
PC (37:4)	0.00000%	0.00500%	0.00000%	0.02645%	0.00000%	0.00084%
PC (38:1)	0.00000%	0.00371%	0.00000%	0.00309%	0.00000%	0.00109%
PC (38:2)	0.01208%	0.00266%	0.00237%	0.00265%	0.00076%	0.00124%
PC (38:3)	0.00000%	0.00000%	0.00000%	0.00000%	0.00000%	0.00000%

PC (38:4) A	0.00000%	0.00052%	0.00000%	0.00080%	0.00000%	0.00036%
PC (38:4) C	0.00000%	0.00177%	0.00000%	0.00595%	0.00000%	0.00250%
PC (38:5) A	0.00852%	0.00058%	0.00218%	0.00124%	0.00090%	0.00062%
PC (38:5) B	0.00000%	0.00023%	0.00000%	0.00053%	0.00000%	0.00040%
PC (38:6) C	0.00000%	0.00030%	0.00000%	0.00059%	0.00000%	0.00040%
PC (p-34:1) or PC (o-34:2) A	0.00000%	0.00000%	0.00000%	0.00000%	0.00000%	0.00000%
PC (p-34:2) or PC (o-34:3)	0.00000%	0.00000%	0.00000%	0.00000%	0.00000%	0.00000%
PC (p-38:4)/PC (o-38:5) B	0.00000%	0.00148%	0.00000%	0.00293%	0.00000%	0.00180%
PC (p-38:5)/PC (o-38:6) A	0.00000%	0.00032%	0.00000%	0.00130%	0.00000%	0.00058%
PC (p-38:6)/PC (o-38:7)	0.00000%	0.00026%	0.00000%	0.00074%	0.00000%	0.00051%
PC (p-40:6)/PC (o-40:7) B	0.00000%	0.00054%	0.00000%	0.00078%	0.00000%	0.00043%
PC (p-40:7) or PC (o-40:8)	0.00000%	0.00000%	0.00000%	0.00058%	0.00000%	0.00000%
PE (34:1)	0.01376%	0.00000%	0.00296%	0.00000%	0.00027%	0.00000%
PE (34:2)	0.01690%	0.00000%	0.00430%	0.00000%	0.00141%	0.00000%
PE (35:1); PE (17:0-18:1);	0.00000%	0.00000%	0.00000%	0.00000%	0.00000%	0.00000%
PE (35:2); PE (17:1-18:1);	0.00000%	0.00000%	0.00000%	0.00000%	0.00000%	0.00000%
PE (36:1)	0.01091%	0.00000%	0.00283%	0.00000%	0.00093%	0.00000%
PE (36:2)	0.00000%	0.00000%	0.00000%	0.00000%	0.00000%	0.00000%
PE (36:3)	0.00276%	0.00000%	0.00156%	0.00000%	0.00027%	0.00000%
PE (38:2)	0.00258%	0.00000%	0.00070%	0.00000%	0.00023%	0.00000%
PE (38:2); PE (18:1-20:1);	0.00000%	0.00000%	0.00000%	0.00000%	0.00000%	0.00000%
PE (p-34:2) or PE (o-34:3)	0.00000%	0.02494%	0.00000%	0.00000%	0.00000%	0.00487%
PE(34:2)	0.00000%	0.00061%	0.00000%	0.00053%	0.00000%	0.00052%
PE(36:1)	0.00000%	0.00071%	0.00000%	0.00060%	0.00000%	0.00051%
PE(36:2)	0.00000%	0.00140%	0.00000%	0.00115%	0.00000%	0.00128%
PE(38:4)	0.00000%	0.00031%	0.00000%	0.00068%	0.00000%	0.00075%
PE(38:6)	0.00000%	0.00000%	0.00000%	0.00039%	0.00000%	0.00024%
PG (34:1); PG (16:0-18:1);	0.00000%	0.00000%	0.00000%	0.00000%	0.00000%	0.00000%
PG (34:2); PG (16:0-18:2);	0.00000%	0.00000%	0.00000%	0.00000%	0.00000%	0.00000%
PG (36:1); PG (18:0-18:1);	0.00000%	0.00000%	0.00000%	0.00000%	0.00000%	0.00000%
PG (36:2); PG (18:1-18:1);	0.00000%	0.00000%	0.00000%	0.00000%	0.00000%	0.00000%
PG (36:3); PG (18:1-18:2);	0.00000%	0.00000%	0.00000%	0.00000%	0.00000%	0.00000%
SM (d30:1)	0.00549%	0.00000%	0.00124%	0.00000%	0.00059%	0.00000%
SM (d41:1)	0.00363%	0.00000%	0.00051%	0.00000%	0.00023%	0.00000%
SM (d42:1)	0.00399%	0.00000%	0.00083%	0.00000%	0.00030%	0.00000%
SM(d33:1)	0.00000%	0.00009%	0.00000%	0.00010%	0.00000%	0.00008%
SM(d36:3)	0.00000%	0.00012%	0.00000%	0.00010%	0.00000%	0.00007%
SM(d38:2)	0.00000%	0.00024%	0.00000%	0.00012%	0.00000%	0.00008%
SM(d40:0)	0.00000%	0.00024%	0.00000%	0.00000%	0.00000%	0.00007%
SM(d43:1)	0.00000%	0.00000%	0.00000%	0.00007%	0.00000%	0.00006%
TG (44:2)	0.00000%	0.00483%	0.00000%	0.00000%	0.00000%	0.00896%
TG (46:0)	0.02278%	0.00000%	0.05026%	0.00000%	0.00652%	0.00000%
TG (46:2)	0.02482%	0.00638%	0.02035%	0.00374%	0.00568%	0.00796%
TG (46:3) A	0.00000%	0.00068%	0.00000%	0.00046%	0.00000%	0.00032%
TG (46:3) B	0.00000%	0.00068%	0.00000%	0.00046%	0.00000%	0.00034%
TG (47:1)	0.00000%	0.00150%	0.00000%	0.00018%	0.00000%	0.00014%
TG (48:0)	0.17690%	0.00000%	0.23571%	0.00000%	0.04094%	0.00000%
TG (48:1)	0.17225%	0.18264%	0.26058%	0.14498%	0.23155%	0.20957%
TG (48:2)	0.07460%	0.03094%	0.08831%	0.02232%	0.02740%	0.04158%
TG (48:3)	0.03833%	0.00420%	0.00763%	0.00641%	0.00213%	0.00387%
TG (48:4) A	0.00000%	0.00049%	0.00000%	0.00115%	0.00000%	0.00094%
TG (48:4) B	0.00000%	0.00040%	0.00000%	0.00115%	0.00000%	0.00094%
TG (48:5)	0.00000%	0.00073%	0.00000%	0.00049%	0.00000%	0.00054%
TG (48:6)	0.00000%	0.00051%	0.00000%	0.00035%	0.00000%	0.00043%
TG (49:1)	0.07364%	0.07015%	0.06071%	0.04685%	0.78995%	0.70856%
TG (49:1); TG (15:0-16:0-17:1);	0.00000%	0.00169%	0.00000%	0.00051%	0.00000%	0.00104%
TG (49:2)	0.03214%	0.02167%	0.01196%	0.02406%	0.05771%	0.07908%
TG (49:3)	0.00000%	0.00061%	0.00000%	0.00021%	0.00000%	0.00602%
TG (50:0)	0.09157%	0.05268%	0.13085%	0.33008%	0.05450%	0.05138%
TG (50:1)	5.58410%	3.87715%	6.74433%	5.45169%	3.19317%	3.57440%
TG (50:2)	0.77138%	1.73634%	3.52637%	1.81048%	2.79143%	2.69166%
TG (50:3)	0.20578%	0.00000%	0.21477%	0.00000%	0.16931%	0.00000%
TG (50:3) A	0.00000%	0.15796%	0.00000%	0.40701%	0.00000%	0.16415%
TG (50:3) B	0.00000%	0.15582%	0.00000%	0.40306%	0.00000%	0.16122%

TG (50:4)	0.06028%	0.01580%	0.04974%	0.02932%	0.02738%	0.02334%
TG (50:5)	0.00617%	0.00297%	0.00218%	0.00362%	0.00094%	0.00182%
TG (50:6)	0.00000%	0.00228%	0.00000%	0.00225%	0.00000%	0.00081%
TG (51:1)	0.00000%	0.09922%	0.00000%	0.09690%	0.00000%	0.22209%
TG (51:2)	0.12715%	0.21475%	0.18218%	0.20011%	2.69956%	2.64819%
TG (51:2); TG (16:1-17:1-18:0);	0.00000%	0.00116%	0.00000%	0.00051%	0.00000%	0.00036%
TG (51:3)	0.03673%	0.04239%	0.04428%	0.05481%	0.16931%	0.15167%
TG (51:4)	0.02064%	0.01135%	0.01159%	0.01247%	0.04565%	0.04231%
TG (51:5)	0.00000%	0.00389%	0.00000%	0.00349%	0.00000%	0.00488%
TG (52:0)	0.03298%	0.01803%	0.03242%	0.03205%	0.07176%	0.01465%
TG (52:1)	3.27624%	4.56281%	3.30322%	3.14769%	4.54754%	3.81247%
TG (52:1); TG (14:0-18:1-20:0);	0.00000%	0.06647%	0.00000%	0.00740%	0.00000%	0.05797%
TG (52:2)	27.89445%	11.32578%	27.02358%	13.68640%	14.34607%	8.81805%
TG (52:2); TG (16:0-16:0-20:2);	0.00000%	0.05412%	0.00000%	0.09757%	0.00000%	0.07999%
TG (52:2); TG (16:0-18:1-18:1);	0.00000%	0.05956%	0.00000%	0.09757%	0.00000%	0.09114%
TG (52:3)	2.92863%	6.19197%	7.28377%	6.30821%	7.10463%	7.41152%
TG (52:4)	0.70579%	1.69808%	1.79126%	3.00494%	2.47068%	1.86280%
TG (52:5)	0.10706%	0.16274%	0.13131%	0.23179%	0.10977%	0.10003%
TG (52:6)	0.01693%	0.04188%	0.01621%	0.00151%	0.00432%	0.00035%
TG (52:6); TG (16:0-18:3-18:3);	0.00000%	0.04188%	0.00000%	0.08258%	0.00000%	0.00703%
TG (53:1)	0.00000%	0.02490%	0.00000%	0.01451%	0.00000%	0.03002%
TG (53:2)	0.15383%	0.24309%	0.16959%	0.22853%	0.80644%	0.43783%
TG (53:3)	0.08248%	0.11885%	0.13052%	0.23023%	0.50816%	0.53000%
TG (53:4)	0.00000%	0.00165%	0.00000%	0.00337%	0.00000%	0.00391%
TG (53:5)	0.00000%	0.00494%	0.00000%	0.00884%	0.00000%	0.00779%
TG (54:1)	0.80534%	0.11141%	0.53235%	0.02769%	3.31587%	0.07871%
TG (54:2)	8.79467%	9.71712%	5.92368%	6.54602%	15.23026%	11.38488%
TG (54:3)	35.28218%	18.84869%	28.68685%	24.02607%	19.56897%	32.16717%
TG (54:3); TG (18:0-18:1-18:2);	0.00000%	0.07251%	0.00000%	0.11497%	0.00000%	0.15144%
TG (54:4)	3.16291%	6.87107%	3.44923%	7.62215%	8.22743%	8.01480%
TG (54:5)	0.82176%	0.00000%	2.00172%	0.00000%	3.77462%	0.00000%
TG (54:5) A	0.00000%	1.97777%	0.00000%	4.04253%	0.00000%	3.10643%
TG (54:5) B	0.00000%	0.00000%	0.00000%	0.00761%	0.00000%	0.00805%
TG (54:6)	0.24066%	0.00000%	0.20667%	0.00000%	0.21268%	0.00000%
TG (54:6) A	0.00000%	0.18069%	0.00000%	0.34677%	0.00000%	0.16379%
TG (54:6) C	0.00000%	0.00054%	0.00000%	0.00153%	0.00000%	0.00109%
TG (54:7) A	0.00000%	0.05718%	0.00000%	0.11088%	0.00000%	0.02439%
TG (54:9); TG (18:3-18:3-18:3);	0.00000%	0.03146%	0.00000%	0.00689%	0.00000%	0.00000%
TG (55:1)	0.00000%	0.01708%	0.00000%	0.00796%	0.00000%	0.00867%
TG (55:2)	0.00000%	0.01778%	0.00000%	0.01653%	0.00000%	0.02346%
TG (55:3)	0.00000%	0.02634%	0.00000%	0.05293%	0.00000%	0.03470%
TG (55:4); TG (18:1-18:2-19:1);	0.00000%	0.00350%	0.00000%	0.00557%	0.00000%	0.00417%
TG (56:1)	0.19545%	0.19892%	0.17118%	0.04929%	0.37789%	0.07235%
TG (56:2)	0.52992%	1.09835%	0.19968%	0.19837%	1.11922%	0.39396%
TG (56:3)	0.23221%	0.48634%	0.45031%	1.19044%	0.25271%	0.42641%
TG (56:4)	0.02307%	0.06049%	0.00827%	0.07238%	0.00854%	0.03640%
TG (56:5) A	0.01274%	0.01968%	0.00952%	0.05045%	0.00546%	0.01697%
TG (56:5) B	0.00869%	0.01968%	0.00574%	0.05045%	0.00310%	0.01697%
TG (56:5) C	0.00000%	0.00000%	0.00000%	0.00000%	0.00000%	0.00000%
TG (56:6)	0.03872%	0.00201%	0.00857%	0.00064%	0.00388%	0.00048%
TG (56:7)	0.04282%	0.00000%	0.00739%	0.00000%	0.00340%	0.00000%
TG (56:7) A	0.00000%	0.00000%	0.00000%	0.00045%	0.00000%	0.00054%
TG (57:1)	0.00000%	0.01898%	0.00000%	0.02936%	0.00000%	0.02133%
TG (57:2)	0.00000%	0.03948%	0.00000%	0.01748%	0.00000%	0.02026%
TG (57:3); TG (18:1-19:1-20:1);	0.00000%	0.00287%	0.00000%	0.00199%	0.00000%	0.00220%
TG (58:1)	0.05035%	0.04767%	0.15713%	0.04948%	0.15267%	0.04405%
TG (58:2)	0.18934%	0.26435%	0.13724%	0.07796%	0.38330%	0.16090%
TG (58:3)	0.03091%	0.02116%	0.01198%	0.00947%	0.02972%	0.01294%
TG (58:4)	0.00000%	0.00511%	0.00000%	0.00298%	0.00000%	0.00556%
TG (58:4) A	0.02369%	0.00000%	0.01240%	0.00000%	0.01220%	0.00000%
TG (58:5)	0.00000%	0.00377%	0.00000%	0.00254%	0.00000%	0.00215%
TG (58:6)	0.00000%	0.00054%	0.00000%	0.00036%	0.00000%	0.00049%
TG (58:7); TG (18:1-18:1-22:5);	0.00000%	0.00060%	0.00000%	0.00045%	0.00000%	0.00095%
TG (58:8)	0.01261%	0.00000%	0.00294%	0.00000%	0.00101%	0.00000%

TG (59:2)	0.00000%	0.04316%	0.00000%	0.06736%	0.00000%	0.08190%
TG (59:3)	0.00000%	0.00648%	0.00000%	0.00837%	0.00000%	0.01015%
TG (60:1)	0.00000%	0.00848%	0.00000%	0.01113%	0.00000%	0.01180%
TG (60:2)	0.06122%	0.06683%	0.32296%	0.08912%	0.33610%	0.13457%
TG (60:3)	0.00000%	0.00867%	0.00000%	0.00849%	0.00000%	0.01226%
TG (60:4)	0.00000%	0.00224%	0.00000%	0.00335%	0.00000%	0.00450%
TG (62:1)	0.00000%	0.00225%	0.00000%	0.00165%	0.00000%	0.00584%
TG (62:2)	0.00000%	0.00695%	0.00000%	0.01230%	0.00000%	0.01842%
TG (62:3)	0.00000%	0.00102%	0.00000%	0.00191%	0.00000%	0.00254%
TG (62:4)	0.00000%	0.00023%	0.00000%	0.00068%	0.00000%	0.00127%
TG (64:2)	0.00000%	0.00041%	0.00000%	0.00147%	0.00000%	0.00252%
TG (64:3)	0.00000%	0.00041%	0.00000%	0.00033%	0.00000%	0.00038%
Total	100%	100%	100%	100%	100%	100%
